# Electrocardiographic findings in a cross-sectional study of human immunodeficiency virus (HIV) patients in Enugu, south-east Nigeria

**DOI:** 10.5830/CVJA-2016-007

**Published:** 2016

**Authors:** PO Njoku, EC Ejim, BC Anisiuba, SO Ike, BJC Onwubere

**Affiliations:** Department of Medicine, University of Nigeria Teaching Hospital, Ituku-Ozalla, Enugu, Nigeria; Department of Medicine, Federal Medical Centre, Keffi, Nasarawa State, Nigeria; Department of Medicine, University of Nigeria Teaching Hospital, Ituku-Ozalla, Enugu, Nigeria; Department of Medicine, University of Nigeria Teaching Hospital, Ituku-Ozalla, Enugu, Nigeria; Department of Medicine, University of Nigeria Teaching Hospital, Ituku-Ozalla, Enugu, Nigeria; Department of Medicine, University of Nigeria Teaching Hospital, Ituku-Ozalla, Enugu, Nigeria

**Keywords:** abnormalities, electrocardiogram, highly active antiretroviral therapy, human immunodeficiency virus

## Abstract

**Background:**

Electrocardiographic (ECG) abnormalities are prevalent in subjects with human immunodeficiency virus (HIV) infection. In this study, three groups of subjects were investigated and the prevalence of ECG abnormalities was analysed.

**Methods:**

A cross-sectional study was carried out on adults between November 2010 and November 2011 at the University of Nigeria Teaching Hospital, Enugu, Nigeria. One hundred HIV-infected patients on highly active anti-retroviral therapy (HAART), 100 HIV-infected HAART-naïve patients and 100 HIV-negative controls were recruited. Twelve-lead electrocardiograms were done on all subjects. Data were analysed using the chi-squared, Student’s t-, one-way ANOVA and Duncan post hoc tests.

**Results:**

Left-axis deviation was seen in 15 (16%) of the HIV-positive subjects on HAART, 10 (13.7%) of the HAART-naïve subjects and eight (21%) of the controls (p = 0.265). Eight (11%) subjects with left ventricular hypertrophy (p <0.001) and two (2.7%) with ST-segment elevation were found among the HIV-positive HAART-naïve subjects (p = 0.134). Prolonged QTc interval was seen in 17 (18.2%) of the HIV-positive patients on HAART, 12 (16.4%) of the HIV-positive HAART-naïve patients and four (10.5%) of the controls (p = 0.012).

**Conclusion:**

The prevalence of ECG abnormalities was higher in the HIV-positive patients on HAART (93%) and the HIV-positive HAART-naïve patients (73%) compared to the controls.

## Background

The global prevalence of HIV/AIDS in people aged 15–49 years was 0.8% in 2011,^1^ affecting approximately 34 million people. An estimated 23.5 million (22.1–24.8 million) of these people, representing 69% of the global HIV burden, reside in sub-Saharan Africa.^2^ Worldwide, Nigeria has the second highest number of new infections reported each year, and an estimated 3.7% of the population or 3.4 million people are living with HIV infection.^3^,^4^

With increased access to anti-retroviral therapy (ART) in resource-poor countries, longevity has increased among people living with HIV/AIDS.^5^ Indeed, dramatic reductions in morbidity and mortality rates have been noted in these patients since the introduction of ART in 1996. In Nigeria, this has prolonged and improved the quality of life of HIV/AIDS patients, with survival data of 68.3% of adults and children who were on ART in 2009, alive and healthy after 12 months.^6^

Although the use of highly active anti-retroviral therapy (HAART) is associated with virological suppression and immunological recovery in people living with HIV infection, HIV and HAART, especially protease inhibitors, induce disorders of lipid metabolism,^7^,^8^ such as diabetes and dyslipidaemia, which are implicated in the increased incidence of cardiovascular disease in this patient population.^9^,^10^

The electrocardiogram (ECG) identifies abnormalities in HIV/AIDS patients whether or not they were suspected of having cardiac disease.^11^ Barbaro and colleagues found ECG abnormalities, including supraventricular and ventricular ectopic beats, as well as non-specific ST–T-wave abnormalities in about 57% of asymptomatic HIV-infected patients.^12^

Sani and colleagues^13^ documented abnormal ECG findings in 81% of 100 AIDS patients, 65% of 78 HIV-positive asymptomatic subjects and 37.5% of 80 HIV-negative subjects. The ECG abnormalities described included different types of arrhythmias,low-voltage QRS complexes, non-specific ST-segment and T-wave changes, poor R-wave progression, right bundle branch block, axis deviations, enlargement of various heart chambers and QTc prolongation.

Asymptomatic ECG findings specific for myocardial ischaemia (Q waves and ST-segment depression, and T-wave inversion in men) signify an increased risk of myocardial infarction or death in HIV-uninfected adults,^14^ and may have worse consequences in HIV-infected individuals. The ECG, which is fairly widely available in resource-poor countries, is helpful in evaluating HIV subjects for cardiovascular disease.

Considering the huge impact of the HIV/AIDS disease burden in sub-Saharan Africa, increasing access to HAART as well as the improved longevity of people living with HIV/ AIDS, there is a need for proper cardiovascular evaluation of this patient population. Early identification and management of cardiovascular diseases in this population would optimise patient care and provide the much-needed information on this subject in sub-Saharan Africa. This study analysed the ECG findings in HIV-infected individuals, including those on HAART, and HIV-negative subjects residing in Enugu, Nigeria.

## Methods

This cross sectional study was carried out from November 2010 to November 2011 at the University of Nigeria Teaching Hospital (UNTH), Enugu, south-east Nigeria. The procedures followed were in accordance with the ethical standards of the Helsinki Declaration (1964, amended most recently in 2008) of the World Medical Association. This study was approved by the ethics committee of the UNTH, Enugu, and written consent was obtained from the subjects. Information obtained was anonymised as far as possible.

Inclusion criteria were adult Nigerians aged 18 years and older with confirmed HIV-positive serology. HIV screening was by enzyme-linked immunoassay (ELISA) and confirmed by Western-blot electrophoresis, while CD4 T-lymphocytes (CD4 cells) were quantified by flow cytometry.

Sample size was calculated using the Fisher’s formula:15 n = z^2^pq/d^2^ where n = minimum sample size; z = 95% confidence level i.e. 1.96; d = level of precision (0.075)^13^; p = maximum prevalence reported in a study of a similar population^16^ (13.6%); and q = 1–p.

A minimum sample size of 80 was calculated. For the purpose of the study, 100 HIV-positive patients who had not taken HAART were recruited into the HIV-positive, HAART-naïve group. One hundred patients who had received HAART for at least three months were enrolled in the group of HIV-positive patients on HAART. Another 100 controls with already known HIV-negative serology were recruited from those being screened for blood donation, marriage and insurance purposes.

Patients in end-stage AIDS disease, classified as category C by the Centre for Disease Control, 1993, were excluded.^17^ Other subjects excluded were those under 18 years of age, individuals with arterial hypertension, coronary artery disease or active symptoms suggestive of ischaemic heart disease or congestive cardiac failure, cardiomyopathy, peripheral or cerebrovascular disease or diabetes mellitus. Further exclusion criteria were patients who were pregnant or in pueperium, as well as those with a significant history of tobacco and or alcohol use, or those who used drugs known to affect the cardiovascular system.

All subjects were evaluated clinically and anthropometric parameters such as height (m), weight (kg), body mass index (kg/ m^2^) and body surface area (m^2^) were assessed. Qualifying subjects had a resting 12-lead surface ECG recording in the supine position at a speed of 25 mm/s using a two-channel automated Techmel ECG machine (USA), ECG-1101 model.

ECG tracings from each participant were analysed in the standard fashion with the long lead II tracing serving as the rhythm strip. Parameters analysed were heart rate, rhythm, P wave (duration, shape), height (paroxysmal atrial complexes), PR interval, QRS wave (duration, shape, height, axis), paroxysmal ventricular complexes, QT interval, QTc, Q wave, T wave (shape), ST-segment (shape), and R and S waves for ventricular hypertrophy.

Echocardiography was also carried out on each of the subjects using the SonoScape SS1-5000 machine and transducer of frequency 3.5 MHz. M-mode, two-dimensional, pulsed-wave, continuous-wave, tissue Doppler imaging and colour Doppler assessments were done with the subject in the left lateral decubitus position. Measurements were taken (in cm) using the American Society of Echocardiography guidelines (leading-edge methodology).^18^

## Statistical analysis

Statistical analysis of data was done using EPI INFO version 6 software. The chi-squared test was used to test the association between categorical variables. Continuous variables were analysed using the Student’s t-test. Comparison of mean ± standard deviations of parameters across the three groups was done using one-way ANOVA, and the Duncan *post hoc* multiple comparison test was done to indicate means for groups in homogenous subsets (means not significantly different) (Table 1). A p-value < 0.05 was taken as statistically significant.

**Table 1:** Comparison of mean ± standard deviations of parameters across the three groups using one-way ANOVA

*Parameters*	*HIV-positive on HAART*	*HIV-positive HAART-naïve*	*Control*	*F-value*	*p-value*
Age (years)	35.85 ± 8.94	34.43 ± 9.49	35.76 ± 9.74	0.716	0.490
Weight (kg)	65.77 ± 13.92*	62.40 ± 12.45	68.69 ± 8.67*	7.007	0.001
Height (m)	1.66 ± 0.07*	1.66 ± 0.09*	1.71 ± 0.79	17.886	< 0.001
BMI (kg/m^2^)	24.14 ± 4.55*	22.47 ± 3.65	24.18 ± 3.32*	6.301	0.002
BSA (m^2^)	1.77 ± 0.17*	1.75 ± 0.18*	1.81 ± 0.15	4.420	0.013
Heart rate (bpm)	82.92 ± 14.08*	84.28 ± 16.79*	68.77 ± 8.02	40.232	< 0.001

*Duncan post hoc multiple comparison test indicating means for groups in homogenous subsets (means not significantly different). BMI = body mass index, BSA = body surface area.

## Results

Three hundred adults were recruited for the study, comprising the group of 100 HIV-positive patients on HAART, made up of 49 males and 51 females, 100 HIV-positive HAART-naïve patient group, made up of 48 males and 52 females, and 100 apparently healthy adults (control group), made up of 52 males and 48 females. There was no significant difference in the gender distribution of these three groups (χ^2^ = 0.347, p = 0.841) (Table 2).

**Table 2:** Gender distribution of the study groups

*Groups*	*Male, n (%)*	*Female, n (%)*	*Total, n (%)*
HAART	51 (51.0)	49 (49.0)	100 (100)
HAART-naive	48 (48.0)	52 (52.0)	100 (100)
Control	52 (52.0)	48 (48.0)	100 (100)
Total	151 (50.3)	149 (49.7)	300 (100)

χ^2^ = 0.347, p = 0.841. HAART = highly active antiretroviral therapy.

The mean age of the HIV-positive patients on HAART was 35.85 ± 8.94 years, that of the HIV-positive HAART-naïve patients was 34.43 ± 9.49 years, while that of the control group was 35.76 ± 9.74 years. There was no significant difference in the mean age of the three groups (F = 0.72, p = 0.49). There was no significant difference in the age groups of the patients and controls (χ^2^ = 4.74, P = 0.19) (Table 3).

**Table 3:** Age distribution in the study groups

*Age(years)*	*HIV-positive on HAART, n (%)*	*HIV-positive HAART-naïve, n (%)*	*HIV-negative control, n (%)*	*Total, n (%)*
<26	11 (23.9)	19 (41.3)	16 (34.8)	46 (100)
26–30	22 (37.9)	19 (32.8)	17 (29.3)	58 (100)
31–35	21 (30.0)	24 (34.3)	25 (35.7)	70 (100)
36–40	17 (35.4)	16 (33.3)	15 (31.3)	48 (100)
41–45	13 (46.4)	7 (25.0)	8 (28.6)	28 (100)
46–50	9 (31.0)	9 (31.0)	11 (37.9)	29 (100)
> 50	7 (33.3)	6 (28.6)	8 (38.1)	21 (100)
Total	100 (33.3)	100 (33.3)	100 (33.3)	300 (100)

χ^2^ = 4.739, p = 0.192. HIV = human immunodeficiency virus; HAART = highly active antiretroviral therapy.

The mean duration of HAART medication for the HIV-positive patients on HAART was 4.0 ± 2.4 years, with minimum and maximum durations of one and 10 years, respectively. Of these patients, 7% took the HAART regimen containing protease inhibitors (PIs), while 93% took HAART that did not contain PIs. However, those on PIs received it for less than six months.

Table 4 shows ECG abnormalities in the study groups and controls. T-wave inversion (< 3 mm) in leads V1–V3 (anterior leads) was the commonest abnormality in all study groups. It was seen in 44 (65.7%) of the HIV-positive subjects on HAART, 22 (45.8%) of the HIV-positive HAART-naïve subjects and 14 (29.2%) of the controls. T-wave inversion (< 3 mm) in leads II, III and aVF (inferior leads) was also seen in two (2.2%) HIV-positive subjects on HAART, one (1.4%) HIV-positive HAART-naïve subject, and six (15.8%) control subjects.

**Table 4:** ECG abnormalities in the study population

*ECG abnormalities*	*HIVpositive on HAART, n (%)*	*HIVpositive HAART naïve, n (%)*	*Control, n (%)*	*χ^2^*	*p-value*
LAD	15 (16)	10 (13.7)	8 (21)	2.656	0.265
T wave inversion in leads V1 –V3	44 (47)	22 (30.4)	14 (36.8)	24.682	< 0.001
Low QRS voltage complex	1 (1.1)	0 (0)	0 (0)	2.007	0.367
1st-degree heart block	3 (3.2)	1 (1.4)	2 (5.3)	1.020	0.600
T-wave inversion in leads II, III, aVF (inferior leads)	2 (2.2)	1 (1.4)	6 (15.8)	4.811	0.090
VEB	1 (1.1)	1 (1.4)	0 (0)	2.007	0.367
T-wave inversion in leads I, aVL,V5–V6 (lateral leads)	0 (0)	2 (2.7)	2 (5.3)	2.027	0.363
LBBB	1 (1.1)	0 (0)	0 (0)	2.007	0.367
RBBB	1 (1.1)	0 (0)	2 (5.3)	2.020	0.364
LVH	0 (0)	8 (11)	0 (0)	16.438	< 0.001
Sinus tachycardia	8(8.6)	14 (19.2)	0 (0)	2.020	0.364
ST-segment elevation	0 (0)	2 (2.7)	0 (0)	4.027	0.134
Sinus bradycardia	0 (0)	0 (0)	2 (5.3)	25.000	< 0.001
Mean QTc ± SD	0.42 ± 0.04	0.41 ± 0.04	0.39 ± 0.03	*15.779	< 0.001
Prolonged QTc	17 (18.2)	12 (16.4)	4 (10.5)	8.784	0.012
Total	93 (100)	73 (100)	38 (100)		

*F-value. For QTc, F = 15.779; p < 0.001. Duncan post hoc multiple comparison test showed all significantly different.

LAD = left axis deviation, VEB = ventricular ectopic beat, LBBB = left bundle branch block, RBBB = right bundle branch block, LVH = left ventricular hypertrophy.

Left-axis deviation (LAD), that is, QRS axis of < 0° to 90°, was seen in 15 (16%) of the HIV subjects on HAART, 10 (13.7%) of the HAART-naïve subjects and eight (21%) of the controls. First-degree heart block (PR interval > 0.2 s) was seen in three (3.2%) HIV-positive subjects on HAART, one (2.1%) HIV-positive HAART-naïve subject, and two (5.3%) control subjects.

One (1.1%) subject with low QRS-wave voltage complex (amplitude of QRS complex < 5 mm in limb leads or > 10 mm in precordial leads), left bundle branch block (LBBB), and right bundle branch block (RBBB), respectively, was found in the group of HIV-positive subjects on HAART, while eight (11%) subjects with left ventricular hypertrophy (LVH) and two (2.7%) with ST-segment elevation were found among the HIV-positive HAART-naïve subjects.

Sinus tachycardia (heart rate > 100 beats per minute) was seen in eight (8.6%) HIV-positive subjects on HAART, and 14 (19.2%) control subjects, while sinus bradycardia (heart rate < 60 beats per minute) was seen in only two (5.3%) of the control subjects. Prolonged QTc (QTc > 0.44 in males or > 0.46 in females) was seen in 17 (18.2%) of the HIV-positive patients on HAART, 12 (16.4%) of the HIV-positive HAART-naïve patients and four (10.5%) of the control subjects.

## Discussion

The prevalence of ECG abnormalities was higher among HIV-positive patients on HAART (93%) and HIV-positive HAART-naïve patients (73%), compared to the controls (38%) (Table 4). Okoye^19^ found a similar prevalence of ECG abnormalities in 80% of AIDS patients with CD4 cell counts < 200 cells/mm^3^, 60% of HIV-positive subjects with CD4 cell counts > 200 cells/mm^3^ and 35% of HIV-negative healthy controls.

The prevalence of ECG abnormalities found in both HIV-positive patients on HAART and HIV-positive HAARTnaïve patients in this study also compared favourably with the rate of 86% reported by Mouanodji et al.^20^ Conversely, the prevalence of ECG abnormalities in this study was significantly higher than the 53% found by Levy et al.^21^and the 55% documented by Herst et al.^22^ The difference may have been due to the small sample sizes of 32 and 21 patients in the studies done by Levy et al. and Herst et al., respectively. Also, not matching the numbers in the study groups, as well as inclusion of patients at advanced stages of AIDS by Levy et al. could have been reasons for the discrepancy.

LAD, T-wave inversion in leads V1–V3 and prolonged QTc were the three most common ECG abnormalities found in HIV-positive patients on HAART and HIV-positive HAARTnaïve patients, respectively, in this study (Table 4). LAD and T-wave inversion in leads II, III, avF and V1–V3 were the three most common ECG abnormalities in the controls. LAD was found more often in both HIV-positive patients on HAART and HIV-positive HAART-naïve patients than in the control subjects. T-wave inversion in leads V1–V3 was the commonest ECG abnormality in all the groups, occurring in 47% of the HIV-positive patients on HAART, 30.4% of the HIV-positive HAART-naïve patients and 36.8% of the controls. It also occurred in smaller proportions in leads II, III, avF, I and avL. T-wave inversion may signify ischaemia but is often a non-specific finding, especially in women.^23^ No relationship between ECG abnormalities and gender was found in the study.

ECG evidence of asymptomatic ischaemic heart disease (IHD) (Q wave or ST-segment depression) was not found in any of the study groups, although ST-segment elevation was seen in leads V2–V3 in two of the HIV-positive HAARTnaïve patients. Early repolarisation abnormality, commonly seen in blacks,^24^,^25^ may be an explanation for this. In a similar study involving 4 831 HIV-positive adults, including those with hypertension and diabetes mellitus, T-wave inversion was observed in 11.1% of participants and it was substantially more common in women than men. Shikuma et al. found ECG evidence of asymptomatic IHD in 10.9% of participants with no known IHD.^23^ Although the exact reason is not known, a higher occurrence of myocarditis, coronary artery vasculitis and ischaemia,^26^ which cause cardiac abnormalities in HIV-positive patients, may explain this higher prevalence.

Sinus tachycardia was seen more often in the HIV-positive groups (8.6% in HIV-positive patients on HAART, 19.2% in HIV-positive HAART-naïve patients) compared to 0% in the controls. This may have been due to inter-current febrile illness, anaemia, myocarditis and increased metabolic demand in HIV patients.^27^ These conditions induce autonomic dysfunction and increased sympathetic activity. The higher prevalence of sinus tachycardia in HIV-positive HAART-naïve patients may have been due to their higher immunosuppression and the prevalence of these identified factors. Sinus bradycardia, noted in 2% of the controls and 0% of the HIV-positive patients, may have been due to increased vagal tone, commonly seen in healthy young people.^28^

First-degree heart block was found in both patients and controls, although slightly more often in patients than in the control group. Although currently out of phase, febrile illness such as leptospirosis, which occurs commonly in HIV-positive patients, has been reported to cause complete heart block.^29^ The use of HAART, especially those containing PIs, in HIV infection is known to cause prolonged QRS duration, firstdegree atrioventricular block and complete bundle branch block. The conduction abnormalities seen in this study were not unexpected since most of the patients had recurrent fever, and were often not evaluated for leptospirosis. Moreover, some of the HIV-positive patients on HAART in this study were on PIs. Charbit et al.^30^ reported a higher number of patients showing prolonged QRS duration, first-degree atrioventricular block and complete bundle branch block in those taking PIs.

Left ventricular hypertrophy (LVH) assessed by Sokolow and Lyon criteria, as well as Araoye’s voltage criteria,^31^ was found in eight (11%) of the HIV-positive HAART-naïve patients but in none of the HIV-positive patients on HAART or the controls. Four HIV-positive patients on HAART, four HIV-positive HAART-naïve patients and one control subject had LVH, derived with Devereux’s formula.

We found left ventricular mass was increased in HIV infection (Table 5). In similar studies to ours, Barbaro et al.^32^ and Lipshultzet al.^33^ reported increased LV mass in HIV-positive patients. On the other hand, Martinez-Garcia et al.^34^ found decreased LV mass in asymptomatic HIV-infected patients, and Samaan et al.^35^ found decreased LV mass among patients with AIDS wasting syndrome.

**Table 5:** Comparison of echocardiographic parameters measured across the groups using one-way ANOVA

*Parameters*	*HIV-positive on HAART*	*HIV-positive HAART-naïve*	*Control*	*F-value*	*p-value*
AO (cm)	2.71 ± 0.40*	2.41 ± 0.37	2.74 ± 0.42*	21.363	< 0.001
LA (cm)	3.27 ± 0.62	2.68 ± 0.51	3.11 ± 0.47	31.385	< 0.001
EDD (cm)	4.73 ± 0.70*	4.41 ± 0.55	4.75 ± 0.42*	11.240	< 0.001
ESD (cm)	3.01 ± 0.51	2.84 ± 0.57	2.92 ± 0.43	2.616	0.075
IVS (cm)	0.77 ± 0.17*	0.85 ± 0.17	0.78 ± 0.15*	6.098	0.003
PW (cm)	0.82 ± 0.16	0.87 ± 0.17	0.82 ± 0.13	2.878	0.058
EF (%)	68.95 ± 12.43*	72.81 ± 11.70	67.36 ± 9.04*	6.223	0.002
FS (%)	36.77 ± 9.81	36.51 ± 8.64	37.77 ± 6.53	0.623	0.537
LVM (g)	141.94 ± 49.75	138.61 ± 48.53	131.26 ± 31.55	1.540	0.216
LVMI (g/m^2^)	79.95 ± 26.25	77.55 ± 25.91	72.37 ± 16.52	2.760	0.065

*Duncan post hoc multiple comparison test indicating means for groups in homogenous subsets (means not significantly different).

AO = aorta; LA = left atrium; EDD = end-diastolic diameter; ESD = end-systolicdiameter; IVS = interventricular septum; PW = posterior wall of left ventricle; EF = ejection fraction; FS = fractional shortening; LVM = left ventricular mass; LVMI = left ventricular mass index.

The mechanisms by which these adverse effects on LV mass occur in HIV-positive patients are not fully understood, but are thought to be related to mitochondrial toxicity.^36^ Many studies have shown that HIV virions directly affect myocardial cells and are associated with local release of cytokines and other factors leading to inflammation, myocarditis and dilated cardiomyopathy.^37^ Also, increase or decrease in LV mass has been suggested to be associated with opportunistic infections andmalnutrition,^35^ therefore a lower nadir CD4 cell count has been associated with higher LV mass index. Meng et al.^38^ reported greater interventricular septal and posterior wall thicknesses among patients exposed to PIs compared to those who were not exposed.

The finding of higher numbers of HIV-positive patients with LVH in our study was not surprising, given the pathogenesis and sequela of HIV infection as well as the use of HAART. Pewsner et al. and Devereux found LVH, assessed by ECG, only in HIV-positive HAART-naïve patients, probably because of the high specificity and low sensitivity of the ECG.^39^,^40^ Onthe other hand, Michael found four patients with LVH, using echocardiography, in both HIV-positive patients on HAART and the HIV-positive HAART-naïve group, and one patient in the control group. This may have been because echocardiography is much more sensitive than ECG.^41^

QTc interval, corrected for heart rate using Bazett’s formula, was more prolonged in our HIV-positive patients on HAART and HIV-positive HAART-naïve patients than in the controls (Table 4). The prevalence of prolonged QTc was 34.6% in HIV-positive patients and 10.5% in the controls. A breakdown of this showed a higher prevalence of 18.2% in HIV-positive patients on HAART, compared to 16.4% in the HIV-positive HAART-naïve group. This is similar to the 45% reported by Okoye22 and 34.7% reported by Ogunmodede.^42^

Villa et al.^43^ reported a high prevalence of prolonged QTc interval of 65% in a highly selected cohort of HIV-positive patients who had already developed autonomic dysfunction. On the other hand, Kocheril et al.^44^ reported a prevalence of 29% in 42 AIDS patients in the absence of any known cause. QTc prolongation in HIV-positive patients has been attributed to electrolyte imbalance from poor nutrient intake and recurrent diarrhoea, drugs including zidovudine,^45^ protease inhibitors,^46^ pentamidine,^46^ halofantrin,^47^ trimethoprim-sulfamethoxazole,^48^ and autonomic dysfunction due to HIV-associated neuropathy.^43^

Okoye^14^ documented hypocalcaemia as the cause of QTc prolongation in 35% of AIDS patients. In our study, the higher QTc prolongation in the patients may have been dueto electrolyte imbalance from vomiting and diarrhoea, PIs, zidovudine, trimethoprim-sulfamethoxazole and antimalarial medications. The higher prevalence seen in HIV-positive patients on HAART, compared to the HIV-positive HAART-naïve group may have been from the use of lopinavir and zidovudine medications.

There were some limitations of this study. First, we were unable to carry out biochemical tests such as serum electrolytes and lipograms in any of the subjects. Second, we were unable to determine the actual time of HIV infection, and by extension, the duration of HIV infection. Third, since this was an observational, cross-sectional study, we were unable to infer causality.

## Conclusion

The prevalence of ECG abnormalities was higher in the HIV-positive patients on HAART (93%) and HIV-positive HAART-naïve patients (73%) compared to the apparently healthy controls. The use of ECG is helpful in cardiovascular evaluation of this patient population, especially in resource-poor countries.
